# The ketamine metabolite (*2R,6R*)‐hydroxynorketamine rescues transcriptional pathways involved with immune activation and mRNA translation in APP/PS1 mice

**DOI:** 10.1002/alz70855_099460

**Published:** 2025-12-23

**Authors:** Joao D Calixtro, Felipe C. Ribeiro, Danielle Cozachenco Ferreira, Rubens L Soares‐Neto, Fernanda Guarino De Felice, Mychael V. Lourenco, Ottavio Arancio, Argel Aguilar‐Valles, Nahum Sonenberg, Sergio T. Ferreira

**Affiliations:** ^1^ Federal University of Rio de Janeiro, Rio de Janeiro, Rio de Janeiro, Brazil; ^2^ Federal University of Rio de Janeiro, Rio de Janeiro, Brazil; ^3^ Institute of Biophysics Carlos Chagas Filho, Federal University of Rio de Janeiro, Rio de Janeiro, Rio de Janeiro, Brazil; ^4^ Queen's University, Kingston, ON, Canada; ^5^ D'Or Institute for Research and Education, Rio de Janeiro, RJ, Brazil; ^6^ Universidade Federal do Rio de Janeiro, Rio de Janeiro, Brazil; ^7^ Department of Medicine ‐ Columbia University, New York, NY, USA; ^8^ Carleton University, Ottawa, ON, Canada; ^9^ McGill University, Montreal, QC, Canada

## Abstract

**Background:**

Hippocampal mRNA translation (i.e., protein synthesis) is crucial for synaptic plasticity and memory consolidation, and becomes defective in AD. We investigated here whether the ketamine metabolite HNK could rescue transcription profiles related to mRNA translation in aged APP/PS1 mice.

**Method:**

Mice were treated with HNK (0.5 mg/kg, i.p.) or saline daily for 14 days, and hippocampal transcriptomic changes were assessed by RNA‐seq. These data were then analyzed by gene ontology (GO) enrichment and reactome analysis.

**Result:**

GO analyses revealed significantly upregulated pathways in APP/PS1 mice (compared to WT mice) that were corrected by HNK treatment. These included regulation of programmed cell death and response to hormones and stress. Reactome pathway analyses further implicated the innate immune system and, notably, three pathways associated with RNA metabolism and translation that were aberrantly regulated in APP/PS1 mice and were rescued by HNK.

**Conclusion:**

Altogether, these results indicate that HNK rescues transcriptional programs associated with inflammation, impaired proteostasis, calcium signaling, and synaptic proteins in aged APP/S1 mice.

